# The Epidemiological Particularities of Malignant Hemopathies in French Guiana: 2005–2014

**DOI:** 10.3390/cancers16112128

**Published:** 2024-06-03

**Authors:** Mathieu Nacher, Qiannan Wang, Beatrice Cenciu, Alolia Aboikoni, Florin Santa, Fabrice Quet, Fanja Vergeade, Antoine Adenis, Nathalie Deschamps, Kinan Drak Alsibai

**Affiliations:** 1 CIC INSERM 1424, Centre Hospitalier Andrée Rosemon, Cayenne 97300, French Guiana; alolia.aboikoni@ch-cayenne.fr (A.A.); fabrice.quet@ch-cayenne.fr (F.Q.); antoine.adenis@ch-cayenne.fr (A.A.); nathalie.deschamps@ch-cayenne.fr (N.D.); 2 Département Formation Recherche Santé, Université de Guyane, Cayenne 97300, French Guiana; 3 Amazonian Institute for Population Health, Cayenne 97300, French Guiana; 4 Registre des Cancers de Guyane, Centre Hospitalier Andrée Rosemon, Cayenne 97300, French Guiana; qiannan.wang@ch-cayenne.fr (Q.W.); kdrak.alsibai@doctor.com (K.D.A.); 5 Hôpital de Jour Adultes, Centre Hospitalier Andrée Rosemon, Cayenne 97300, French Guiana; beatrice.cenciu@ch-cayenne.fr; 6 Service d’Hépatogastroentérologie, Centre Hospitalier Andrée Rosemon, Cayenne 97300, French Guiana; 7 Service de Médecine, Centre Hospitalier Andrée Rosemon, Cayenne 97300, French Guiana; florin.santa@ch-cayenne.fr; 8 Service de Santé Publique, Centre Hospitalier de l’Ouest Guyanais, Saint Laurent du Maroni 97320, French Guiana; f.vergeade@ch-ouestguyane.fr; 9 Centre de Ressources Biologiques Amazonie, Centre Hospitalier de Cayenne, Cayenne 97300, French Guiana; 10 Department of Pathology, Centre Hospitalier Andrée Rosemon, Cayenne 97300, French Guiana

**Keywords:** malignant hemopathies, multiple myeloma, adult t-cell lymphoma, HTLV-1, incidence, mortality, Latin America

## Abstract

**Simple Summary:**

French Guiana has a high prevalence of HIV and HTLV-1; its population is ethnically mixed, with widespread poverty, and up to 20% of the population lives in geographic isolation. These singular conditions suggest that the epidemiology of hematological malignancies may also be unusual. We, therefore, studied the incidence and mortality of different hematological malignancies and described factors associated with case fatality. The standardized incidence rate for hematological malignancies was 32.9 per 100,000 male years and 24.5 per 100,000 female years. Multiple myeloma and adult t-cell lymphoma/leukemia due to HTLV-1 infection were the two most common hematologic malignancies and causes of death. People born in a foreign country had a poorer case-fatality rate, presumably reflecting difficulties in accessing care. The epidemiology of hematological malignancies in French Guiana has features that distinguish it from mainland France or from Latin America.

**Abstract:**

French Guiana is a French Overseas territory with singular features: it has a high prevalence of HIV and HTLV-1, its population is ethnically mixed, with widespread poverty, and up to 20% of the population lives in geographic isolation. In this context, we used registry data to estimate incidence and mortality due to hematological malignancies and to compare them with France and tropical Latin America. ICD codes C90 and C88 were compiled between 2005 and 2014. The direct standardization of age structure was performed using the world population. Survival analysis was performed, and Kaplan–Meier curves were drawn. The overall standardized incidence rate was 32.9 per 100,000 male years and 24.5 per 100,000 female years. Between 2005 and 2009, the standardized incidence rate was 29.6 per 100,000 among men and 23.6 per 100,000 among women, and between 2010 and 2014, it was 35.6 per 100,000 among men and 25.2 per 100,000 among women. Multiple myeloma/plasmocytoma and mature t/NK cell lymphomas, notably adult t-cell lymphoma/leukemia due to HTLV-1 infection, were the two most common hematologic malignancies and causes of death. Non-Hodgkin’s lymphoma incidence estimates were greater than global estimates. After adjusting for age, sex, and type of malignancy, people born in a foreign country independently had a poorer case-fatality rate, presumably reflecting difficulties in accessing care. The epidemiology of hematological malignancies in French Guiana has features that distinguish it from mainland France or from Latin America. The incidence of multiple myeloma and adult t-cell lymphoma/leukemia was significantly greater in French Guiana than in France or other Latin American countries.

## 1. Introduction

French Guiana lies between Brazil and Suriname. This young French territory has an ethnically mixed population, with the majority of individuals of African ancestry. Although its GDP per capita is the highest in Latin America, over half of the population is poor. Its health expenditure per capita is the highest in Latin America [1], and despite low health professional density, it has connections with large cancer centers in France where patients are often referred for treatment. For patients treated in French Guiana, all approved drugs as part of French cancer treatment protocols are available. French Guiana also has a cancer registry that allows the production of reliable indicators of cancers, which is an important asset to improve knowledge of the distinct local epidemiology. An epidemiologic feature of French Guiana is its high HIV prevalence (>1% for over 30 years) and the high HTLV-1 prevalence in populations of African descent [2,3,4,5,6,7]. These viruses may impact the epidemiologic profile of hematological malignancies in the territory relative to other areas where HIV and HTLV-1 prevalences are lower than in French Guiana [7,8]. Furthermore, HHV8 is prevalent in French Guiana and has also been associated with hematologic malignancies such as multicentric Castelman’s disease (MCD), primary effusion lymphoma, MCD-related immunoblastic/plasmablastic lymphoma, and various atypical lymphoproliferative disorders [9,10,11,12], Another feature distinguishing French Guiana from France is its ethnic makeup, with 64.5% of the population being from African descent [13]. Different studies have suggested that African ancestry was associated with a lower incidence of leukemia but greater mortality than other ethnic groups [14,15,16,17]. We have recently shown that multiple myeloma and plasmocytoma incidence is particularly high, occurring at ages younger than in mainland France, with an unusual predominance of female patients [18].

In this particular context, conflating a high prevalence of exposure in relation to hematologic malignancies and populations with different baseline genetic risks, the objective of the present study was to give an overview of incidence and mortality due to hematological malignancies and to compare them with France and tropical Latin America countries.

## 2. Methods

### 2.1. The Cancer Registry

Since its creation in 2005, the Registre du Cancer de Guyane (RCG) has conducted a regular and as exhaustive as possible inventory of the epidemiology of cancer in French Guiana [19]. The registry compiles data from hundreds of notification and information sources, the vast majority of which are outside French Guiana. Within French Guiana, data collection mostly focuses on the hospitals of Cayenne, Kourou, and Saint Laurent du Maroni. The registration and coding of tumors by research technicians comply with the rules of the networks of French and European registers, FRANCIM (France Cancer Incidence et Mortalité) and ENCR (European Network of Cancer Registries) [20].

Regardless of the cancer location, the registry continuously and exhaustively lists new cases of cancer diagnosed from 1 January 2003, corresponding to invasive and/or in situ tumors of patients residing in French Guiana.

The registry relies on the National Information Systems Medicalization Program data (PMSI), transmitted by FRANCIM and ATIH (Agence technique de l’information sur l’hospitalisation). It mentions all anonymized hospital stays with ICD 10 (10th International Classification of Diseases) codes and a corresponding location code in French Guiana; this allows the registry to query French health structures within and outside of French Guiana. Data collection also relies on local physicians, Medical Information Departments, and hospital archives for access to medical records of patients with cancer, biologists, pathologists, and non-government organizations involved in cancer screening. The registry database is sent each year to the Hospices Civils de Lyon (HCL) and to the International Agency for Research on Cancer (IARC).

### 2.2. Regrouping of Diagnoses

In France, the diagnosis of lymphoma is made by the local pathology department, and then the case is systematically transmitted to a group of national experts called Lymphopath, supported by the National Cancer Institute (INCa). The latter confirms the diagnosis and classifies lymphoma according to the European classification of lymphomas. This diagnosis is the one retained for patient care and by the cancer registry. Some cases of lymphoma remain unclassifiable after the opinion of the Lymphopath expert group (difficult diagnosis or insufficient material for additional techniques), and thus, the diagnosis retained is malignant lymphoma/leukemia of non-specific type. The cancer registry aims to understand the epidemiology of malignant tumors and only records the main types of malignant hematopoietic diseases in its database without including histological subtypes. Therefore, the registry does not have data on subtypes of Hodgkin’s lymphoma or Mantle cell lymphoma.

Given the large number of diagnoses, we regrouped them into a smaller number of groups in two ways. First, we used the international consensus classification [21], and then, in order to compare this with a recent burden of hematologic malignancies paper, we created four categories of leukemia, Hodgkin’s lymphoma, non-Hodgkin’s lymphoma, and multiple myeloma [22].

### 2.3. Analysis

For the present analysis, we searched for tumors using the ICD-O3 classification of diseases.

Data were analyzed with Stata (Stata Corporation, College Station, TX, USA). Direct standardization on age structure was performed using the world population. Because the population of French Guiana is small and hematological malignancies are relatively rare events, we refrained from using year-by-year data, which could yield wide fluctuations, and pooled all cases for the entire period divided by the mean population. For temporal comparisons, because of the yearly fluctuations, we divided this into the following two periods: 2005–2009 and 2010–2014. For case fatality, we used STATA’s st-commands with the origin being the date of inclusion, failure being the date of death, and right censoring referring to the date of the last news given for patients alive or last case of contact.

Kaplan–Meier curves were drawn, and Cox proportional hazards modeling was used. The proportionality of hazards was checked visually and using Schoenfeld residuals. Statistical significance was set at 5%.

### 2.4. Ethical and Regulatory Aspects

The registry received approval from the National authorities (Commission Nationale Informatique et Libertés) and complies with the European General Data Protection Regulation (GDPR).

## 3. Results

The median age was 54.4 years (IQR = 41.2–66.9 years). The M/F sex ratio was 1.3, and 40.8% of patients were born abroad.

Overall, hematologic malignancies were the second most frequent malignancy after prostate cancer. The overall standardized incidence rate was 32.9 per 100,000 male years and 24.5 per 100,000 female years. Between 2005 and 2009, the standardized incidence rate was 29.6 per 100,000 among men and 23.6 per 100,000 among women, and between 2010 and 2014, it was 35.6 per 100,000 among men and 25.2 per 100,000 among women.

**Figure 1:**
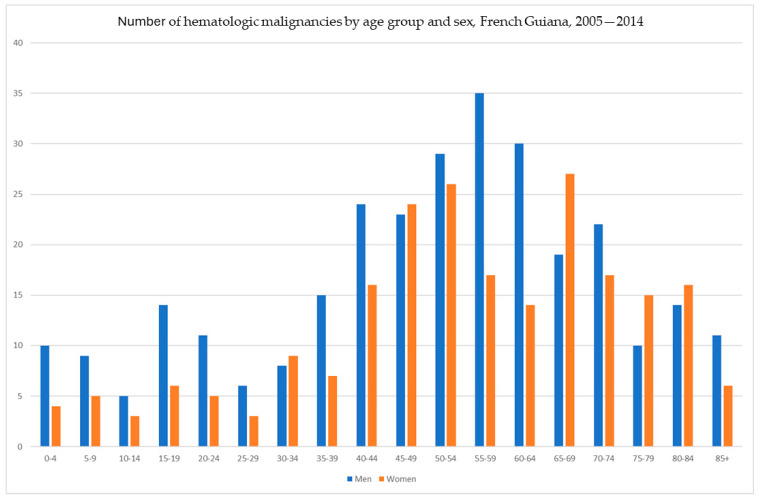
Incidence of hematologic malignancies by age and sex.

**Figure 2:**
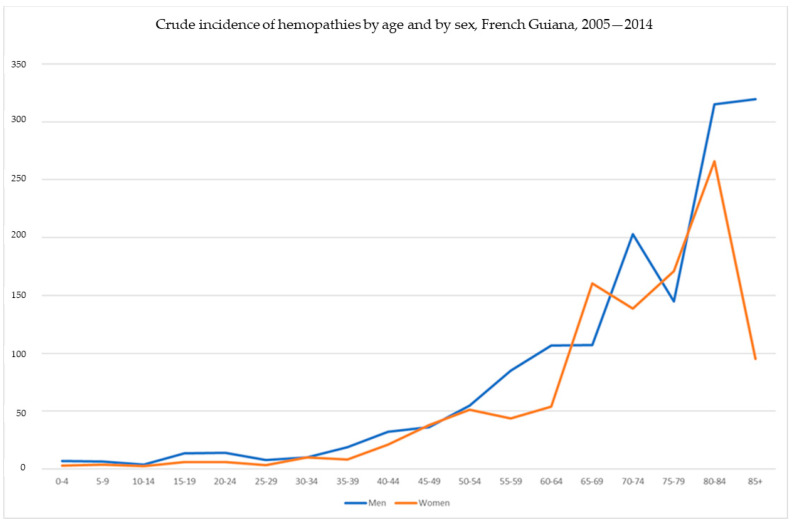
Crude incidence of hematologic malignancies by age and sex.

**Figure 3:**
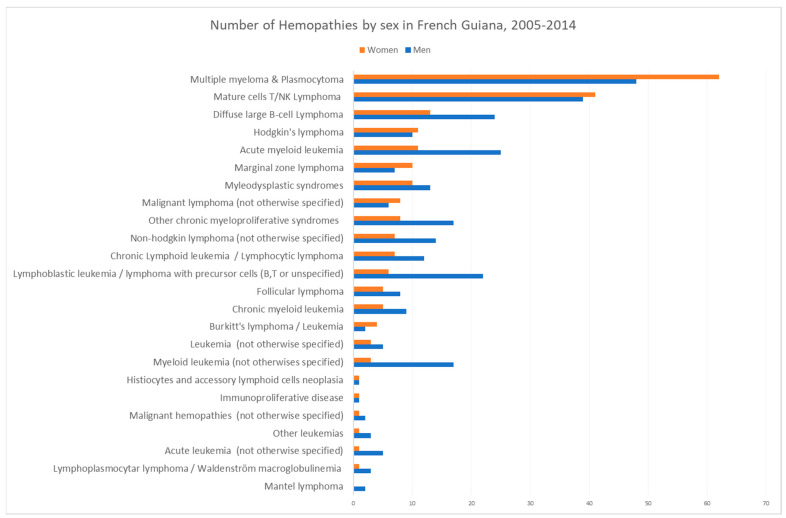
The diagnoses that were identified.

Figure 1 shows the incidence breakdown by age and sex, and Figure 2 shows the crude incidence by age and sex. Figure 3 lists the different diagnoses that were made.

### 3.1. Multiple Myeloma

Between 2005 and 2014, there were 110 cases of multiple myeloma/plasmocytoma (in 62 women and 48 men (Figure 1)), representing the eighth most frequent malignancy in French Guiana and the most frequent hematologic malignancy. This cancer was much more common in the female population. Details on this particular malignancy have been published elsewhere [18].

### 3.2. Mature T/NK-Cell Lymphoma

There were 39 cases—annual age-standardized incidence rate = 4.3 per 100,000—among men and 41—annual age-standardized incidence rate = 4.5 per 100,000—among women during the 2005–2014 period.

For the above mature t/NK-cell lymphoma, a substantial proportion were proven to be HTLV-1 positive, including 21 men (53.8%)—annual age-standardized incidence rate = 2.4 per 100,000—and 18 women (43.9%)—with the annual age-standardized incidence rate = 1.9 per 100,000.

### 3.3. Adult T-Cell Lymphoma/Leukemia

**Figure 4:**
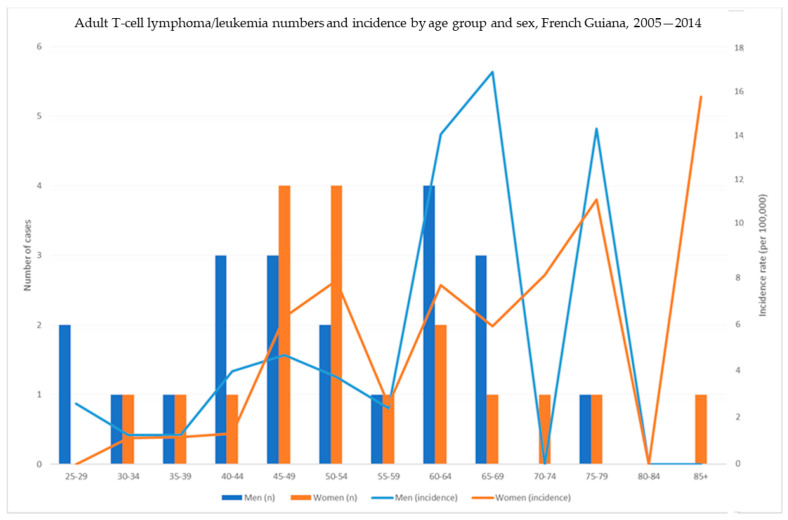
Adult T-cell lymphoma/leukemia: number of cases by age and sex.

The median age at diagnosis for ATL was 51 years among men and 52.5 years among women. Figure 4 shows the incidence and case numbers for age and sex.

#### 3.3.1. Cofactors

Overall, 49 people (10.2%) were HTLV1-positive, and 45 (9.5%) were HIV-positive.

**Figure 5:**
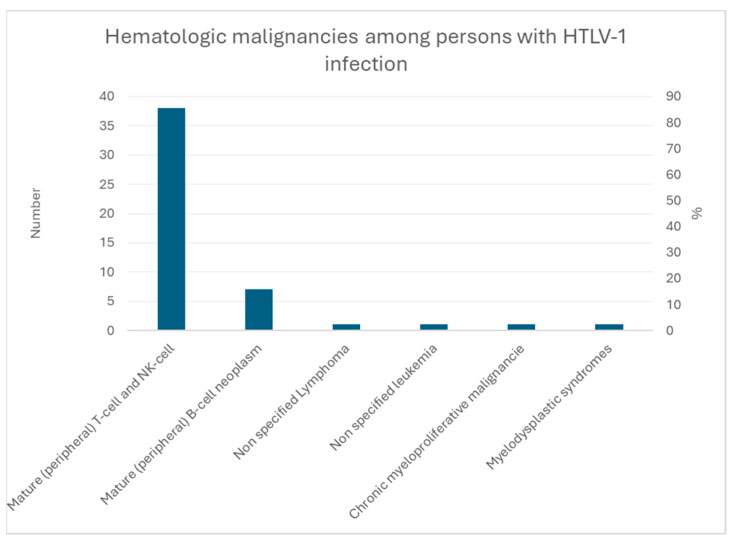
Hematologic malignancies among individuals with HTLV-1 infection.

None of the HTLV-1-positive individuals were born in mainland France, and most were either foreigners (N = 29) or born in French Guiana (N = 20). For HIV, 29 were foreigners, 9 were born in French Guiana, 2 were from another overseas territory, and 2 came from mainland France. Figure 5 shows malignant hemopathies among people living with HTLV-1, mostly for adult t-cell lymphoma/leukemia.

**Figure 6:**
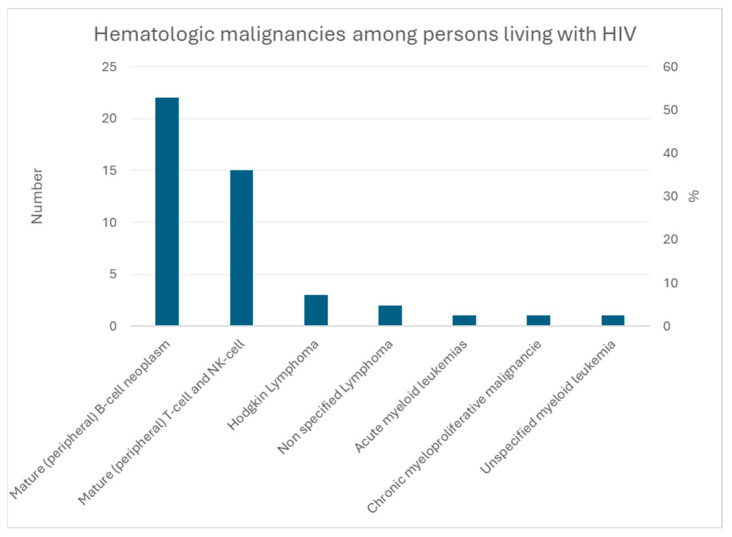
Hematologic malignancies among people living with HIV.

Figure 6 shows malignant hemopathies among people living with HIV, mostly B-cell lymphoma.

#### 3.3.2. Mortality

In total, between 2005 and 2014, 81 men and 54 women with malignant hemopathies died. We assume that most died from their malignant hemopathy because, for the 186 cases where the cause of death was available, 94.6% died from the malignancy. The crude incidence rates were 7.2 per 100,000 man years and 4.7 per 100,000 woman years (overall 6 per 100,000 person years). The standardized incidence rates were 10.2 per 100,000 man years and 6 per 100,000 woman years, respectively (overall 8 per 100,000 person years).

**Figure 7:**
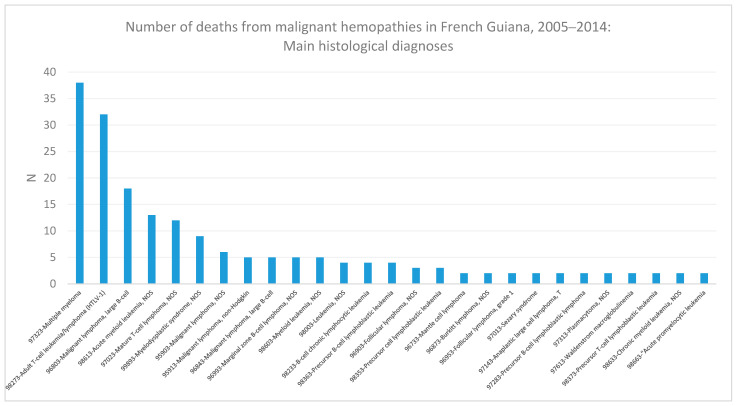
Number of deaths from malignant hemopathies in French Guiana, 2005–2014: main histological diagnoses.

When looking at the main contributors to diagnoses between 2005 and 2014 (Figure 7), the two main causes of death were multiple myeloma/plasmocytoma and HTLV-1-associated t-cell leukemia/lymphoma.

#### 3.3.3. Standardized Incidence and Mortality Rates

**Figure 8:**
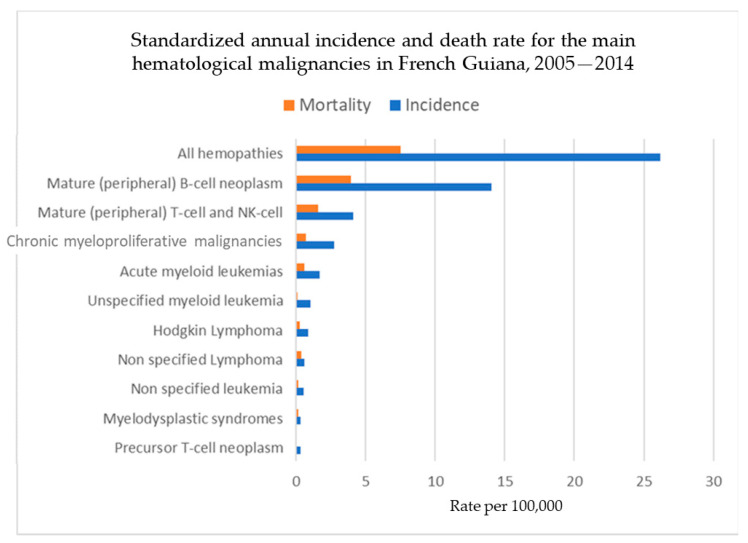
Standardized annual incidence and death rate for the main hematological malignancies in French Guiana, 2005–2014.

Figure 8 shows the standardized incidence and mortality rates for the main hematological malignancies according to the recent International Consensus Classification. Multiple myeloma, mature B-cell lymphoma, and mature T-cell lymphoma had the greatest incidence and mortality.

**Figure 9:**
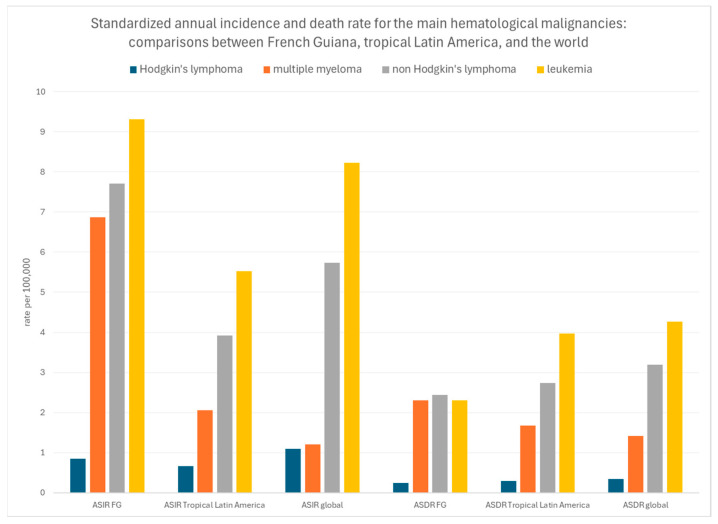
Standardized annual incidence and death rate for the main hematological malignancies: comparisons between French Guiana, tropical Latin America, and the world. ASIR: age-standardized incidence rate; ASDR age-standardized death rate.

Figure 9 uses another classification (in order to align with a recent burden of hematological malignancies study) and shows that standardized incidence rates of multiple myeloma, non-Hodgkin’s lymphoma, and leukemia were relatively high in French Guiana, but, by contrast, that standardized mortality rates seemed lower.

#### 3.3.4. Case Fatality

**Figure 10:**
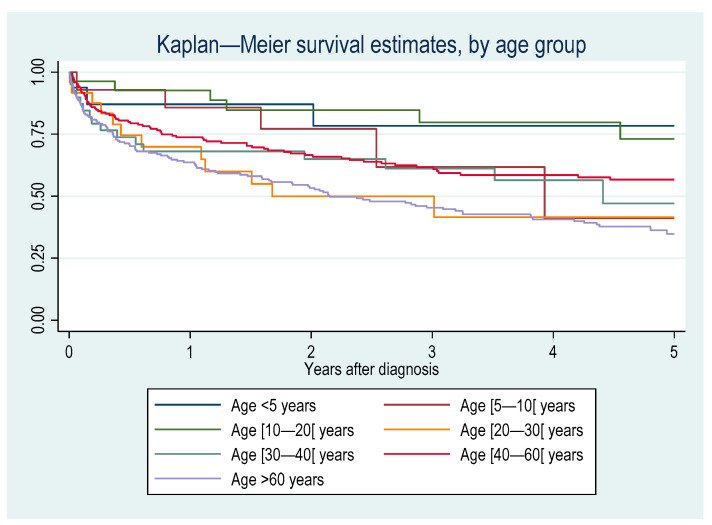
Incidence of death among patients with a hematological malignancy by age group.

**Figure 11:**
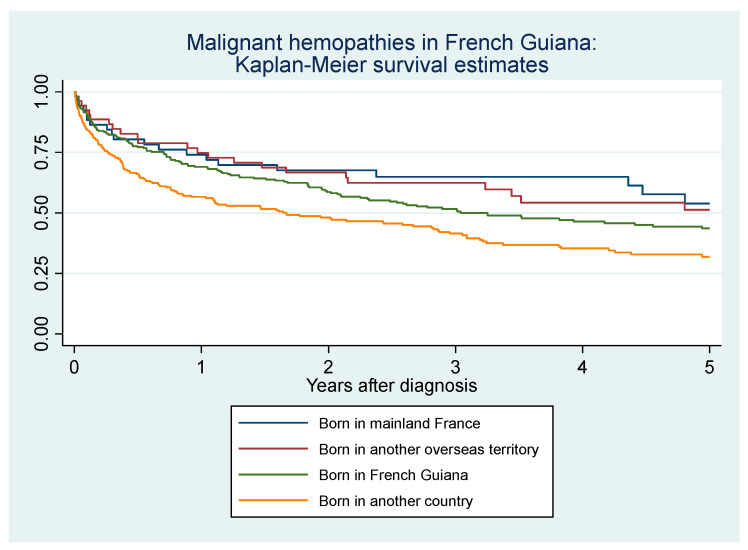
Incidence of death among patients with a hematological malignancy by birthplace.

**Figure 12:**
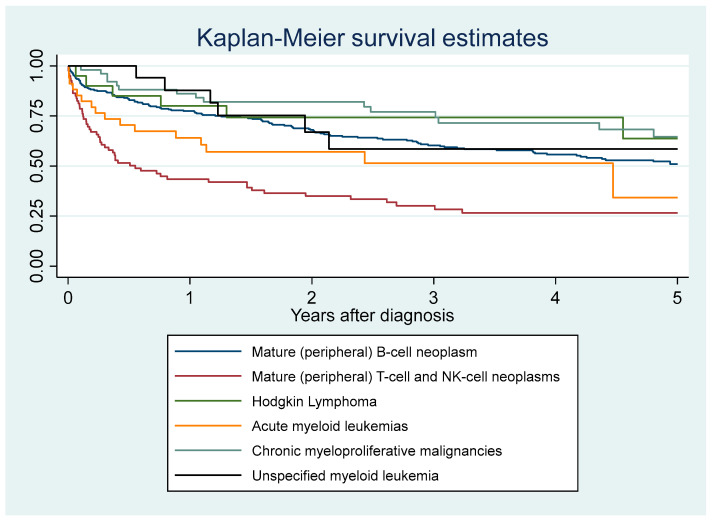
Incidence of death among patients with a hematological malignancy by type of malignancy.

Figure 10 shows the crude incidence of death among patients with a hematological malignancy by age group. Figure 11 shows the crude incidence of death among patients with a hematological malignancy by origin. People living with HIV had an increased hazard of death after adjustments for the type of hemopathy, age, and sex (aHR = 1.5 (95%CI = 1.04–2.3)) but after introducing place of birth (80% of persons with HIV are foreign bord in French Guiana), this was no longer significant. Foreigners and those born in French Guiana died earlier than those born in mainland France or other overseas territories. Individuals living in a village only accessible by boat or air had a significantly greater fatality rate per case than those living in a village accessible by road. Figure 12 shows Kaplan–Meier survival curves for the most frequent diagnoses. Table 1 shows that after adjusting for the type of hemopathy, age, and sex, foreigners had a greater risk of dying than those from mainland France. Mature t-cell and NK-cell neoplasms and unspecified lymphomas had a significantly greater risk of dying than the reference group (mature (peripheral) B-cell neoplasm).

## 4. Discussion

The hypothesis that we aimed to test was whether hematological malignancies in French Guiana were more South American than French. However, what we observed was neither a French pattern nor a South American one. Multiple myeloma/plasmocytoma and mature t/NK cell lymphomas, notably adult t-cell lymphoma/leukemia due to HTLV-1 infection, were the two most common hematologic malignancies and causes of death. They also seemed greater than estimates for tropical Latin America or global estimates.

As detailed in reference [18], the incidence of multiple myeloma is significantly greater in French Guiana than in Western Europe or other Latin American countries, and its epidemiology is unusual—female-biased and occurring at younger ages [22,23,24]. We believe the high observed incidence of multiple myeloma may be explained by the frequent African ancestry in French Guiana. Regarding the greater incidence among women than among men, this increasing temporal trend of incidence may be explained by the high and growing frequency of obesity in French Guiana–notably among women.

Another marked feature of hematological malignancies in French Guiana was the high incidence of mature t/NK cell lymphomas—the second most frequent hematological malignancy—and notably of adult t-cell lymphoma/leukemia in French Guiana. Despite progress [25], this was not unexpected given that the prevalence of HTLV-1 infection among populations of African ancestry in French Guiana remains substantial [6,7,25,26]. While it was the second cause of death among hematological malignancies, its case fatality rate was the highest, with over half of patients dying within a year.

An important finding was that among individuals with hematological malignancies in general, after adjusting for age, sex, and type of malignancy, the case-fatality rate was significantly greater among those born in a foreign country—nearly double that of those born in mainland France. Given the fact that, despite France’s universal health system, for all public health problems, foreign-born patients are usually diagnosed later, more likely to renounce care and to interrupt their follow-up, which was not an unexpected finding [27,28]. In a territory where 29% of the population is foreign, the fact that 40.8% of patients with hematological malignancies are foreigners is not surprising. The crude results suggest that those born in French Guiana have a greater case fatality rate. However, after adjusting for age, sex, origin, and type of malignancy, this difference was no longer significant. This may be explained by the fact that among individuals born in French Guiana, HTLV-1, and HIV are more frequent, and HTLV-1 is associated with the highly lethal ATL. Although numbers were small, unsurprisingly, people living in remote villages not connected by road also independently had a greater case fatality rate than those living in a village connected by road. However, for those living in one of the three cities, there was a non-significant trend of greater case fatality rate than those living in a village connected by roads. Perhaps this should be interpreted as the detrimental impact of isolation –whether geographic or social—on survival.

It also appeared that non-Hodgkin’s lymphoma incidence was also higher than in tropical Latin America or globally. However, adjusted mortality rates were not. This combination of high incidence but low mortality may have resulted from the high HIV prevalence in French Guiana (1.2% overall and 1.6% in the 15–49 years old [29]) combined with a well-funded universal health system [30] offering the latest oncological therapeutic advances to all patients, irrespective of their social condition.

The present study has a number of limitations. First, it is arguable that our numbers are small and pale in comparison to territories with larger populations, notably for the rarest malignancies. However, the registry strives to maximize exhaustivity; thus, if numbers are relatively small, it is because the population of French Guiana is small, not because diagnoses are not reported. Therefore, the incidences and mortality rates are of value for the territory and for the Guiana shield region. Furthermore, despite the relatively small numbers, the survival analyses using Cox regression were still feasible. Another limitation is that one of the diagnoses was not sufficiently precise enough to classify them appropriately. Therefore, this may have led to an underestimation of the actual number of cases of other properly labeled diagnoses. The cause of death is not always easy to ascertain, and thus, perhaps mortality rates and case fatality rates may have been overestimated. However, when the cause of death was available, 94.6% were linked to the hematological malignancy. Furthermore, we believe relative comparisons remain reliable, especially after adjusting for age, sex, and birthplace. The incidence estimates in French Guiana seemed greater than in tropical Latin America or globally, and there are plausible explanations for this, for example, the high HIV or HTLV-1 prevalences. However, French Guiana, as a sparsely populated territory with three hospitals, means that exhaustive collection of cases and deaths may be easier than larger countries with hundreds of hospitals to cover. In this scenario, the observed differences in incidence rates could partly reflect differences in exhaustivity. Another special circumstance of French Guiana is the intense immigration fluxes, and it is conceivable that some patients with hematological malignancies declared a local address (when, in fact, they lived in a neighboring country), thus increasing incidence; furthermore, when there is no hope of a cure, such patients may often end up going back to die home unbeknownst to us. Despite these drawbacks, we still believe that the insights regarding the main malignancies, their case fatality, and the variables that are associated with them are important. Reducing HIV and HTLV-1 transmission and fighting obesity may yield some future benefits.

## 5. Conclusions

The epidemiology of hematological malignancies in French Guiana has features that distinguish it from mainland France or Latin America. The incidence of multiple myeloma was significantly higher in French Guiana than in France or other Latin American countries. In contrast to the usual predominance of multiple myeloma in males, there was a preponderance in females, and the median age was younger in French Guiana than in France. Another striking feature in French Guiana was the high incidence of mature t/NK cell lymphomas, notably adult t-cell lymphoma/leukemia, due to HTLV-1 infection and its very poor prognosis. After adjusting for age, sex, and the type of malignancy, individuals born in a foreign country independently had a poorer case-fatality rate, presumably reflecting difficulties in accessing care. Finally, non-Hodgkin’s lymphoma incidence seemed greater than global estimates, perhaps high HIV prevalence in French Guiana is part of the explanation. The above features suggest that the prevention of obesity or HTLV-1 transmission and the treatment and prevention of HIV transmission may have some benefits on some of the main hematologic malignancies. Improving access to diagnosis and care for the most vulnerable populations may improve prognosis and case fatality in general.

## Figures and Tables

**Table 1:** Adjusted hazard ratios of dying from a hematological malignancy.

	Hazard Ratio	[95% CI]	P
**Age category (years)**			
<5	Reference		
[5–10]	4.58	0.88–23.79	0.07
[10–20]	2.19	0.45–10.75	0.34
[20–30]	4.93	1.10–22.10	0.04
[30–40]	5.30	1.20–23.42	0.03
[40–60]	4.78	1.16–19.67	0.03
>60	8.16	2.00–33.33	0.00
**Hemopathy**			
Mature (peripheral) B-cell neoplasm	Reference		
Precursor T-cell neoplasm	1.90	0.45–8.10	0.39
Mature t-cell and NK-cell neoplasms	2.47	1.81–3.36	0.00
Hodgkin’s lymphoma	1.06	0.51–2.18	0.88
Non-specified lymphoma	4.76	2.14–10.61	0.00
Non-specified leukemia	2.07	0.65–6.54	0.22
Acute myeloid leukemias	1.74	0.99–3.06	0.05
Chronic myeloproliferative malignancies	0.81	0.51–1.29	0.38
Myelodysplastic syndromes	1.49	0.55–4.07	0.44
Unspecified myeloid leukemia	0.76	0.33–1.73	0.51
**Origin**			
French from mainland France	Reference		
French from another overseas territory	1.11	0.59–2.09	0.74
French from French Guiana	1.51	0.90–2.53	0.12
Foreigner	1.94	1.17–3.22	0.01
**Sex**			
Male	Reference		
Female	0.77	0.60–0.99	0.04
**Type of residence**			
Village accessible by paved road	Reference		
City	1.55	0.96–2.51	0.07
Village only accessible by boat or air	2.51	1.16–2.44	0.02

## Data Availability

Upon reasonable request and after approval from the Commission Nationale Informatique et Libertés, data may be shared.
